# Study and Modeling of the Kinetics of the Photocatalytic Destruction of Stearic Acid Islands on TiO_2_ Films

**DOI:** 10.1021/acs.jpcc.3c00952

**Published:** 2023-06-20

**Authors:** Saleh Alofi, Christopher O’Rourke, Andrew Mills

**Affiliations:** School of Chemistry and Chemical Engineering, Queens University Belfast, Stranmillis Road, Belfast BT9 5AG, U.K.

## Abstract

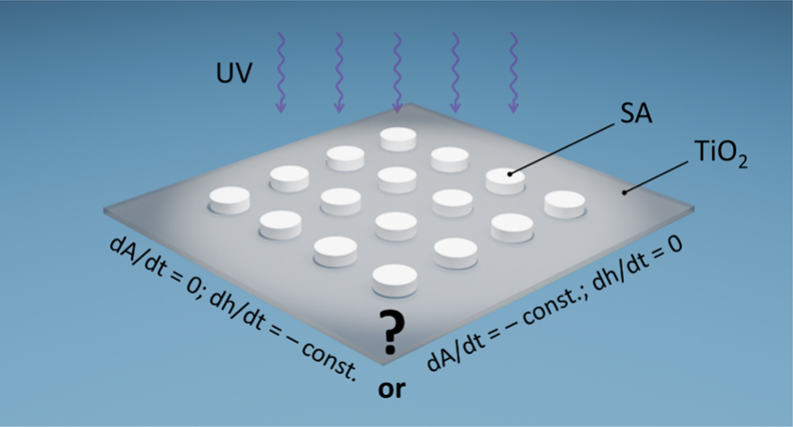

The kinetics of the removal of stearic acid (SA) islands by photocatalytic coatings is controversial, with some reporting that the islands fade as their thickness, *h*, decreases with the irradiation time, *t*, but maintain a constant area, *a*, −d*a*/d*t* = 0, and others reporting that −d*h*/d*t* = 0 and −d*a*/d*t* = −constant, i.e., the islands shrink, rather than fade. This study attempts to understand the possible cause for these two very different observations through a study of the destruction of a cylindrical SA island and an array of such islands, on two different photocatalytic films, namely, Activ self-cleaning glass, and a P25 TiO_2_ coating on glass, which have established uniform and heterogeneous surface activities, respectively. In both cases, using optical microscopy and profilometry, it is shown that, irrespective of whether there is as a single cylindrical island or an array of islands, *h* decreases uniformly with *t*, −d*h*/d*t* = constant, and −d*a*/d*t* = 0, so that the SA islands just fade. However, in a study of the photocatalyzed removal of SA islands with a volcano-shaped profile, rather than that of a cylinder, it is found that the islands shrink and fade. A simple 2D kinetic model is used to rationalize the results reported in this work. Possible reasons for the two very different kinetic behaviors are discussed. The relevance of this work to self-cleaning photocatalytic films is discussed briefly.

## 1 Introduction

At present, the biggest commercial market for photocatalytic products is self-cleaning glass,^1−3^ the active component of which is a thin, ca. 15 nm thick, compact, i.e., non-porous, film of anatase TiO_2_.^4^ Not surprisingly, therefore, an internationally recognized standard for classifying glass as photocatalytic, and self-cleaning, has been developed recently. This test is based on the measurement of the haze of the piece of glass under test that has been initially soiled, before and after irradiation.^5^ A key component of the coating solution used to produce the initial haze on the glass sample is stearic acid (SA). SA is one of the most common saturated fatty acids found in nature and, in research, the photocatalyzed mineralization of SA is one of the most employed reactions used to demonstrate the self-cleaning action of new photocatalytic films.^6−20^ The overall reaction process can be summarized as follows
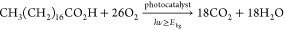
1where *E*_bg_ is the band gap energy of the photocatalyst, which is ca. 3.2 eV for anatase TiO_2_, for example.

In most kinetic studies of the photocatalytic destruction of SA, the whole surface of the photocatalyst is covered with a layer of SA, i.e., a conformal coating of SA. It follows that during its photocatalyzed destruction, the total mass of SA, *m*, in the SA film at irradiation time, *t*, is given by the expression
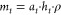
2where *m*_*t*_, *a*_*t*_, and *h*_*t*_ are the values of the mass, area, and thickness, *h*, of the SA film, respectively, at irradiation time *t*, and ρ is the density of SA. In such studies, it is usually found that the area of the SA film does not change during the photocatalytic process, i.e., *a*_*t*_ = *a*_0_, and that the kinetics approximate to zero order with respect to the concentration of SA, [SA]_*t*_ (units: g cm^–2^), at time *t*, where [SA]_*t*_ = *m*/*a*_0_,^4,20−26^ so that the rate of removal of SA is described by the expression
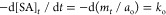
3the integrated form of which is
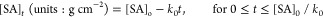
4Where, [SA]_0_ is the initial concentration of SA (=*m*_0_/*a*_0_) and *k*_0_ is the zero-order rate constant which, in turn, is dependent upon the irradiance, φ, of the radiation absorbed by the photocatalyst (units: mW cm^–2^).

The observation of zero-order kinetics for reaction 1 is usually rationalized in terms of the following features: (i) all the photocatalytic sites are occupied by a stack of SA molecules that form an overall film of uniform thickness, *h*, (ii) the rate-determining step is the initial oxidation of SA (i.e., there are no long-lived intermediates), and (iii) all the photocatalytic sites are equally reactive. Note that in the few kinetic studies of this system in which φ has been varied,^26,27^*k*_0_ has been found to depend directly upon φ, which suggests that hole-trapping by the SA is very efficient, which is very likely given (i) and (ii). In some studies of this system, the reaction kinetics are not of zero order and are described by the following expression
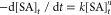
5where *n*, the order of the reaction, is nearer unity.^10,28^ The latter kinetic feature for reaction 1 has been attributed to a distribution of reactivities in the surface sites.^10,28^ Previous work has established that, when the commercial self-cleaning glass product Activ is used as a photocatalytic film for mediating reaction 1, the kinetics are of zero order, i.e., *n* = 0.^10,28^ The latter finding suggests that Activ comprises a surface with a very uniform reaction site photocatalytic activity, which is perhaps not too surprising given its large-scale method of production.^4,28^

In such studies of reaction 1, for a uniform coating of SA on a TiO_2_ film comprising sites of equal activity, in which *n* = 0, it follows that at all *t*, *a*_*t*_ is fixed at its initial value *a*_0_, so that the rate of SA removal, d[SA]/d*t*, can be expressed as follows
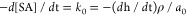
6where −d*h*/d*t* is the rate of loss in the thickness of the SA film. Thus, a physical manifestation of the commonly observed zero-order photocatalyzed decay of [SA], on photocatalytic coatings, is a zero-order decay in SA film thickness, *h*, with a zero-order rate constant of *k*_0_/ρ. However, in practice, in kinetic studies of reaction 1, the variation of the thickness of the SA film as a function of *t* is rarely measured directly. Instead, most studies of reaction 1 are based on the measurement of the integrated absorbance, Abs_int_(*t*), of the FT-IR spectrum of the SA film over the range 2700–300 cm^–1^, which is proportional to [SA]_*t*_, where an integrated absorbance value of 1 cm^–1^ is found to be equivalent to ca. 4.6 μg cm^–2^ of SA.^20−23^

Out in the field, a uniform film of SA on the surface of a photocatalytic self-cleaning product appears to be an unlikely scenario, and that a more likely one is that the surface will, initially at least, become contaminated by islands of the pollutant. However, even in this situation, it would seem reasonable to assume that the destruction of an array of the pollutant islands would proceed along the same lines as those found when the whole of the photocatalytic coating is covered with a film of a pollutant. For example, it would seem reasonable to expect that the overall decay of an array of identical, cylindrical SA islands of the same initial thickness, *h*_0_, dispersed over a photocatalytic coating of uniform activity would exhibit zero-order kinetics with each island showing no change in area *a*, but rather thickness, *h*_*t*_, that decreases linearly with *t*, i.e., −d*a*/d*t* = 0 and −d*h*/d*t* = constant, *c*_*h*_. Evidence that this is indeed the case is provided by the work of Sawunyama et al.^29^ in their study of the photocatalyst destruction of partial monolayers of SA, which formed islands with diameters spanning 1–20 μm. The results of this study revealed that, while the islands showed some evidence of random pitting, due to hot spots of reactivity on the surface of the photocatalyst, there was “no evidence of preferential reaction at island edges compared to the interior regions,”^29^ so that the area of each island remained unchanged during the photocatalytic process, i.e., −d*a*/d*t* = 0, or *a*_*t*_ = *a*_0_ at all *t*. Thus, in the latter work, the SA islands were found to fade with increasing irradiation time, *t*, and not shrink.

**Figure 1:**
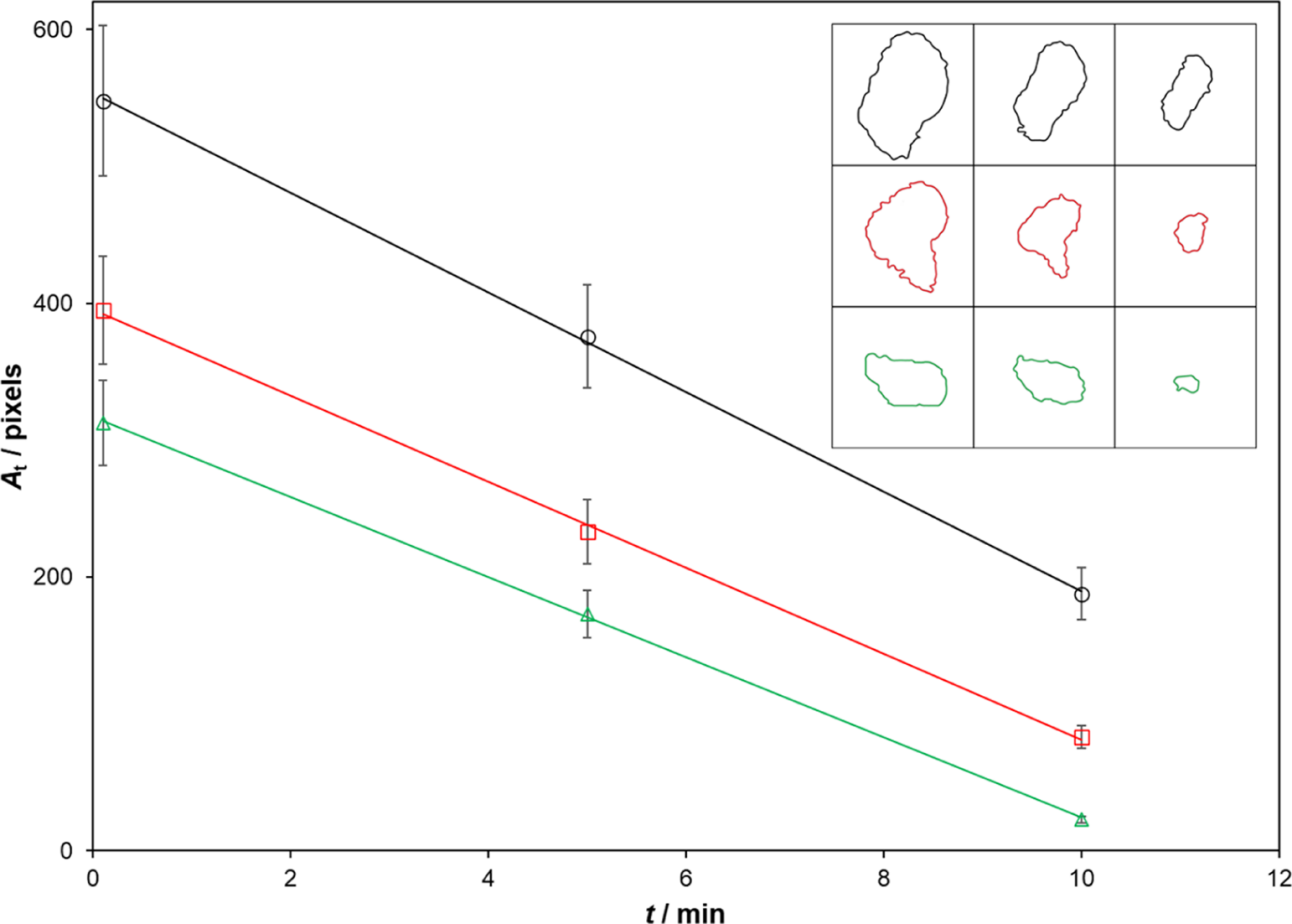
Plot of area (in pixels from optical micrograph) vs irradiation time for three SA islands of similar initial thickness but different initial areas, on a sol–gel TiO_2_ film upon irradiation with 2.5 mW cm^–2^ UVA radiation. Insert diagram illustrates the reported outlines of the three different-sized islands at (from left to right) *t* = 0, 5, and 10 min, respectively.^30^

In striking contrast to the above findings, Ghazzal et al.,^30^ in a study of reaction 1 involving a wide distribution of SA islands on a sol–gel TiO_2_ film, found that *a*_*t*_ decreased at a constant rate with *t*, i.e. −d*a*/d*t* = constant, *c*_a_, and −d*h*/d*t* = 0.^30^ Thus, in the latter work, the SA islands were found to shrink with increasing irradiation time, *t*, and not fade. In this work,^30^ evidence that −d*a*/d*t* = constant, *c*_a_, is provided by, among other things, a plot of the size (in pixels, as assessed by optical microscopy) of three different typical SA islands, all with similar thicknesses, as a function of *t*, as illustrated in Figure 1.^30^ Ghazzal et al. suggest that the reported novel kinetics arise because radical species formed throughout the uncovered TiO_2_ surface diffuse to the edges of the SA islands,^30^ and so, for brevity, we shall refer to this mechanism and its associated kinetics as the edge activity, area dependent (EAAD) model.

The striking discrepancy between the findings of the above two studies^29,30^ and the significance of the subject area when it comes to understanding the self-cleaning action of many photocatalytic products, not least self-cleaning glass, has prompted this study of the kinetics of reaction 1, using primarily a single cylindrical island of SA, and an array of such islands, deposited on a photocatalytic film. In this work, two different types of TiO_2_-based, photocatalytic films were tested, namely one with an established, uniform reactivity, Activ glass, and one with a more heterogenous surface reactivity.

## 2 Materials and Methods

### 2.1 Materials Declaration

Unless stated otherwise, all chemicals were purchased from Merck and used as received. The SA used (Merck, 175366-1KG) was reagent grade with a purity of 95%. Activ self-cleaning glass was supplied by Pilkington Glass-NSG. The Aeroxide P25 TiO_2_ was purchased from Evonik, further details of which are given elsewhere.^31^

### 2.2 Preparation of P25 TiO_2_ Films on Glass

P25 TiO_2_ films on microscope slides were prepared using a drawdown method in which two strips of 3M Scotch Magic tape were placed either side of the microscope slide producing a ca. 2 cm wide, 60 μm deep trough. A P25 TiO_2_ paste was made, comprising 76 mg of P25 TiO_2_ dispersed in a mixed solvent solution made up of 20 mL acetic acid, 20 mL water, 3.98 mL ethanol, 0.43 g terpineol, and 0.74 g of 10% w/w ethyl cellulose in methanol. Further details regarding the preparation of this paste are given elsewhere.^32^ A few drops of the paste were deposited at the top of the 3M Scotch tape trough and drawn down using a glass rod. The resulting 60 μm thick wet film of the paste was then annealed at 450 °C for 1 h (ramp rate = 10 °C min^–1^), to produce the 1.70 μm thick P25 TiO_2_ film used in this study.

### 2.3 Preparation of Sol–Gel TiO_2_ Films on Glass

The preparation of the sol–gel paste is detailed elsewhere.^7,33^ Briefly, 4.65 g of glacial acetic acid were added to 20 mL of the precursor solution, titanium(IV) isopropoxide. 120 mL of deionized water, containing 1.08 g of nitric acid, were then added to the Ti(IV)/acetic acid solution, to produce a white dispersion of the hydrous oxide. This dispersion was used to grow colloidal TiO_2_ particles hydrothermally using an autoclave, 220 °C for 12 h. The resulting white precipitate was then redispersed using an ultrasonic probe (Lucas Dawe Ultrasonics, Soniprobe, London, England), and rotary evaporated until a weight percent of TiO_2_ of 10–12% was achieved, to which 50 wt % of polyethylene glycol was then added. The final white paste was mayonnaise-like in appearance and texture. The paste was then applied to microscope slides using the same drawdown method detailed above. The resulting 60 μm thick wet film of the sol–gel paste was then annealed at 450 °C for 1 h (ramp rate = 10 °C min^–1^), producing a ca. 2.8 μm thick sol–gel TiO_2_ film.

### 2.4 Deposition of Cylindrical SA Islands on Activ

2 mm diameter cylindrical dots of SA were deposited on Activ glass by first dip-coating a 2.5 cm × 7.5 cm slide of Activ in 0.1 M stearic acid in chloroform to produce a uniform coating of SA across the whole of the photocatalytic surface. In the work, the sample of Activ glass was immersed for 20 s into the SA/chloroform solution and then withdrawn at 1000 mm min^–1^ using a KSV NIMA Layer Builder (Biolin Scientific, Gothenburg, Sweden) dip-coater, thereby producing a ca. 150 nm thick film. The back of the resulting SA-coated Activ glass was wiped clean of SA using a cloth soaked in chloroform. An automated cutting machine (Cricut Explore Air 2, South Jordan, USA) was then used to cut 2 mm holes out of 100 μm thick PTFE adhesive tape (3M PTFE Film adhesive Tape 5490), which was then applied to the surface of the SA film covering the Activ glass. The holed PTFE adhesive tape film was pressed flat using a microscope slide to ensure good adhesion to the SA film. The tape was then quickly peeled off, leaving behind an array of 2 mm SA dots, ca. 160 nm thick, on the Activ glass.

### 2.5 Deposition of Cylindrical SA Islands on P25 TiO_2_ Films

2 mm diameter cylindrical dots of SA were deposited on a P25 TiO_2_ film by spray coating, using a Talon TS siphon-feed airbrush (Paasche, TS-3L, Kenosha, USA), 0.1 M stearic acid in chloroform, through a holed acetate template. The template was created by cutting 2 mm circles in the sheet of acetate using an automated cutting machine (Cricut Explore Air 2, South Jordan, USA). The airbrush was held 15 cm from the template, and 5 passes were used to deposit the SA, creating ca. 450 nm thick cylindrical dots.

### 2.6 Deposition of Volcano SA Islands on Sol–Gel TiO_2_ Films

Volcano-shaped islands were deposited on a sol–gel TiO_2_ film using a Talon TS siphon-feed airbrush (Paasche, TS-3L, Kenosha, USA). The airbrush was held parallel to the surface of the TiO_2_ film at a height of 5 cm, and 0.1 M SA in chloroform was sprayed for 5 s, which produced a number of volcano-shaped islands, ca. 50 μm wide and ca. 1 μm tall, on the sol–gel TiO_2_ film.

### 2.7 Irradiations, FT-IR Spectroscopy, Optical Microscopy, and Profilometry

Unless otherwise stated, all irradiations were performed using a 15 W, Analytik Jena UV bench lamp fitted with 254 nm (UVC) bulbs which provided an incident irradiance of 2 mW cm^–2^. The irradiance was measured using a UVX meter (Analytik Jena, Jena, Germany) fitted with a 254 nm sensor. All FTIR spectra were recorded using a Spectrum One FTIR (PerkinElmer, Massachusetts, USA). Digital photographs were taken using a Canon 77D fitted with a Canon EF-S 60 mm f2.8 USM Macro Lens. Optical microscopy was carried out using an Olympus Trinocular Microscope, SZ6045TR (Tokyo, Japan), fitted with a Kiralux 8.9 MP color CMOS camera, CS895CU (ThorLabs, New Jersey, USA). Profilometry measurements were carried out using a Dektak3ST surface profile measuring system (Veeco, California, USA). The SA dots were scanned over a distance of 5 mm at a rate of 100 μm s^–1^, with a stylus force of 3 mg.

Irradiations of the SA volcano islands were performed inside the profilometer. In this work, UVA light (365 nm, ca. 80 mW cm^–2^) was supplied by a 10 W 365 nm LED, ILH-XT01-S365-SC211-WIR200 (Intelligent LED Solutions, Berkshire, UK), through an optical fiber, BFY1000HS02 (ThorLabs, New Jersey, USA), held ca. 2–3 mm above the surface of the SA volcano island. The irradiance was measured using a C10427 UV power meter (Hamamatsu, Shizuoka, Japan).

### 2.8 Photocatalytic Film Characterization

**Figure 2:**
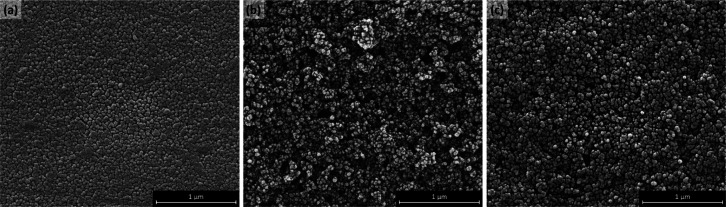
SEM images of the surfaces of (a) Activ glass, (b) P25 TiO_2_ coating, and (c) sol–gel TiO_2_ coating, which reveal TiO_2_ particle sizes of ca. 32, 40, and 44 nm, respectively.

Scanning electron microscopy was carried out using an FEI Quanta FEG - Environmental SEM Oxford Ex-ACT (FEI, Oregon, USA). Figure 2a shows the SEM image of the surface of Activ comprising TiO_2_ particles ca. 32 nm in diameter. As the thickness of the APCVD applied TiO_2_ coating on Activ glass is only ca. 15 nm, the surface consists of tightly packed broad domes of TiO_2_.^4^Figure 2b shows the network of loosely packed interconnected TiO_2_ particles, ca. 40 nm in diameter, of a mesoporous P25 TiO_2_ paste coating, which had a thickness of ca. 1.7 μm.^34^ The SEM of the surface of a sol–gel TiO_2_ coating is shown in Figure 2c, from which it appears that it has a slightly tighter packed and more uniform mesoporous network compared to the P25 TiO_2_ coating, comprising particles ca. 44 nm in diameter. Other work showed that the sol–gel film was ca. 2.8 μm thick.^35^

## 3 2D Kinetic Model

In this work, all the SA islands prepared were symmetrical, usually cylindrical in shape, and so it follows that, instead of modeling the kinetics of the decay of such structures in three dimensions, it is possible to do it equally effectively, and much more simply and quickly, in two dimensions. This section describes a 2D kinetic model that was used to describe the results reported here for a conformal coating of SA (**S1**), a cylindrical island of SA (**S2**), and an array of such islands (**S3**). The basic assumptions of the 2D kinetic model are those usually proposed^4,20−26^ for system **S1**, namely, that all photocatalytic sites are active, only occupied sites are effective, and the kinetics at each site is zero-order with respect to [SA], i.e., saturation kinetics.

### 3.1 General 2D Kinetic Model

The general 2D kinetic model is based on a line of 100 photocatalytic sites, *i*, each with a zero-order rate constant, *k*_*i*_, with an initial uniform covering of SA of thickness, *h*_*i*,0_. Note that the choice of 100 sites is arbitrary, with other work showing that identical model predictions are generated if the number of sites is increased to 1000 or more. In the 2D general kinetic model, it follows from eq 2 that at each reaction site, *i*, the initial concentration of SA will be
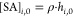
7where *h*_*i*,0_ is the initial thickness of the SA film at zero irradiation time, *t*, so that the initial, total concentration of SA, [SA]_T,0_, will be
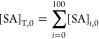
8

Assuming each site, *i*, exhibits zero-order kinetics for reaction 1, it follows from eq 4 that at irradiation time, *t*, the total SA concentration will be
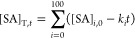
9At this point, it is helpful to define a unitless time parameter, τ
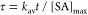
10where *k*_av_ is the average zero-order rate constant, and [SA]_max_ is the maximum initial concentration of SA among all the 100 SA-occupied sites, which is associated with a maximum SA thickness, *h*_max_. Combining eqs 8–10, it is possible to derive the following expression for *f*_SA,τ_, the fractional (or relative) total concentration of SA as a function of τ, i.e.
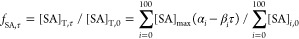
11where α_*i*_ = [SA]_*i*,0_/[SA]_max_ and β_*i*_ = *k*_*i*_/*k*_av_. Note that due to the nature of zero-order kinetics, in carrying out the summations eqs 9 and 11, when the calculated term in parenthesis is negative, then, in the summation, its value is returned as zero.

Equation 11 of the above, 2D general kinetic model of reaction 1 is able to describe the kinetics of reaction 1, when the system is complicated by: (i) having variable amounts SA, i.e., where α_*i*_ varies with *i*, and/or (ii) sites of variable activity, i.e., where β_*i*_ varies with *i*. Note that the use of a dimensionless fractional parameter such as *f*_SA*,*τ_ could hide light-related effects associated with the rate of absorption of the incident UV irradiance, φ. For example, effects such as reflection, scattering, and absorption could change during a reaction and so affect the rate. Fortunately, in this work, both the TiO_2_ films and SA coatings were largely non-scattering, and so, such light-related effects would most likely be minimal.

### 3.2 Simplified 2D Kinetic Model

As noted earlier, most of the work described here was focused on three simple systems, namely, SA deposited as either: **(S1)** a conformal film, **(S2)** a cylindrical island, or **(S3)** an array of cylindrical islands. In addition, since most work was carried out on Activ glass, it was assumed that all sites are equally active. As a result, the kinetics of SA removal for systems **S1–S3**, can be described by the following simplified version of eq 11
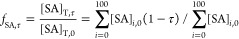
12since, at all sites *i*, *k*_av_ = *k*_*i*_ = *k*_0_ and [SA]_max_ = [SA]_*i*,0_, i.e., α_*i*_ = β_*i*_ = 1. The above equation is for the simplified 2D kinetic model, whereas eq 11 is for the general 2D kinetic model.

Thus, if we assume that each of the 100 photocatalytic sites (of equal activity) are coated with a SA film of initial thickness, *h*_*i*,0_, the value of which is 100 (arbitrary units), then, from eq 9, given [SA]_*i*,*t*_ is proportional to *h*_*i*,*t*_, the variation in the thickness of SA covering each site *i*, as a function of irradiation time τ, will be given by the following expression
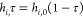
13

**Figure 3:**
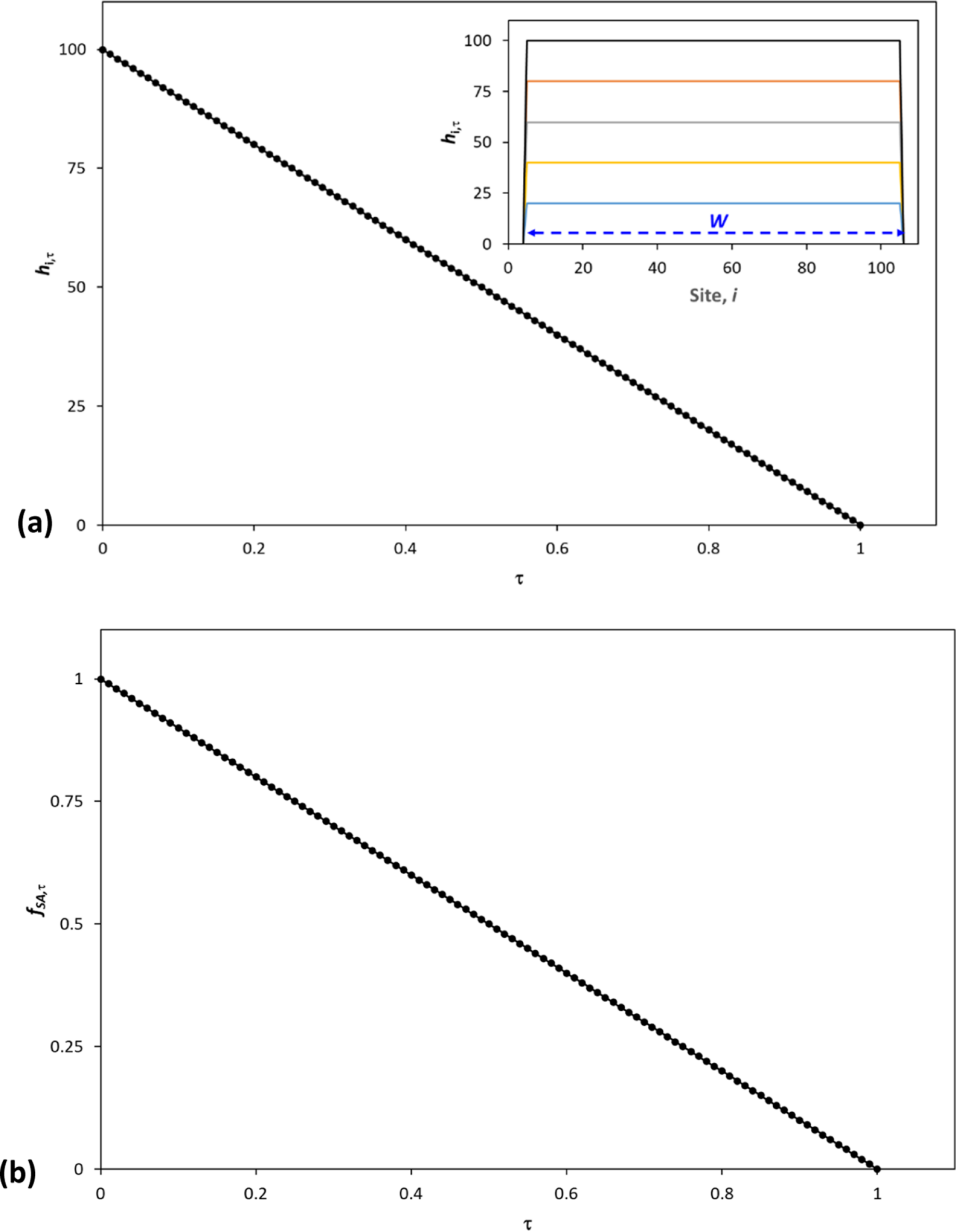
2D kinetic model-predicted plots, for a 2D sheet of SA covering 100 identical (*k*_*i*_ = *k*_0_) photocatalytic sites, with each site, *i*, initially loaded with SA with *h*_*i*,0_ = 100 (a.u.), of: (a) (insert) SA film thickness, *h*_*i*,τ_, vs site number, *i*, and (main diagram) *h*_*i*,τ_ vs τ, calculated using eq 13 of the 2D model; in the insert plot, the different calculated profiles correspond to τ values of (from top to bottom) 0, 0.2, 0.4, 0.6, and 0.8, respectively, and (b) variation in the fraction of SA, *f*_SA,τ_ calculated using eq 12 of the 2D model, vs τ.

Using eq 11, the simplified 2D kinetic model was used to calculate the *h*_*i*,τ_ vs site number, *i*, profiles at different irradiation times, τ, (insert diagram in Figure 3a), as well as the *h*_*i*,τ_ vs τ profile (main diagram), illustrated in the main diagram in Figure 3a. These results show that the simplified 2D kinetic model predicts that the thickness of the SA film, *h*_*i*,_τ, will decrease uniformly with irradiation time, τ, whereas the width of the film, *W*, will remain unchanged. The simplified 2D model can be used to predict the variation in *f*_SA*,*τ_ as a function of irradiation time, τ, using eq 12, and the results of this work are illustrated in Figure 3b. The latter show that the kinetics of destruction of the 2D SA film is zero order, as expected, given all sites are covered initially with the same amount of SA, and so the same *h*_0_, and have the same zero-order rate constant, *k*_0_.

The results illustrated in Figure 3 were generated using the simplified 2D kinetic model for a line of 100 columns/stacks of SA, of initial thickness 100 (a.u.), with each column/stack covering a different photocatalytic site, *i*, all with the same activity, i.e., *k*_*i*_ = *k*_0_. However, as noted earlier, for reasons of symmetry, the kinetics features predicted by this simplified 2D kinetic model, as illustrated in Figure 3, will also be the same for a 3D system comprising a photocatalytic coating of uniform activity, with a SA deposit that is in the form of a conformal film **(S1)**, a cylindrical island **(S2)**, or an array of cylindrical islands **(S3)**. Illustrations of the model-related SA cylindrical island and array of such islands are given in Figure S1a,b, respectively, in the electronic Supporting Information file that accompanies this paper.

Thus, given τ is proportional to irradiation time *t*, the results in Figure 3 show that the simplified 2D kinetic model predicts, for all examples of **S1–S3**, on Activ, the following common kinetic features: (a) no change in the SA film area with *t*, i.e., −d*a*/d*t* = 0, (b) a linear decrease in SA film thickness with *t*, with a gradient -*k*_0_/(ρ·*h*_0_), i.e., −d*h*/d*t* = constant (*c*_*h*_), and (c) zero-order decay kinetics, i.e., *n* = 0, so that a plot *f*_SA,*t*_ vs *t* will be a straight line with a negative gradient, −*k*_0_/[SA]_0_. Table 1 provides a summary of these predictions, which highlights that with increasing irradiation time, in all cases, the SA film/island/islands will fade (−d*h*/d*t* = *c*_*h*_, and NOT shrink, d*a*/d*t* = 0).

**Table 1:**
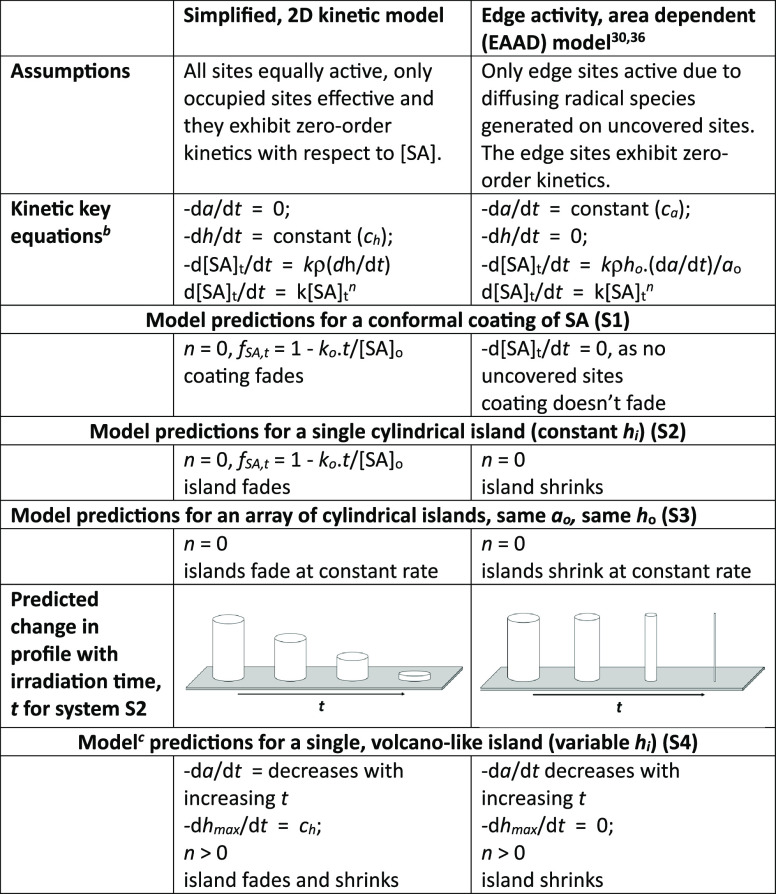
SA Island Photocatalytic Decay Models and Predictions^a^

a Where the island is cylinder shaped, of height *h* and area *a*, and ρ is the density of SA; at *t* = 0, *a* = *a*_0_ and *h* = *h*_0_ and *n* = order of reaction.

b *k* = rate constant, which depends upon the irradiance, φ.

c Using the general 2D kinetic model, with α_*i*_ = 1 and β_*i*_ varied so as to create volcano-shaped initial profile.

In this work, the 2D kinetic model is used exclusively to explain the fading Langmuir–Blodgett SA islands reported by Sawunyama et al.^29^ and the results of numerous unique experiments, involving cylindrical islands and array of such islands and volcano-shaped islands—all of which are consistent with current theory and the model assumptions that all sites are equally active, only occupied sites are effective, and each site exhibits zero-order kinetics with respect to [SA]. Because of its very different underlying assumptions, the 2D kinetic model is not, and cannot be, used to predict the kinetics of the EAAD kinetic model proposed by Ghazzal et al.^30^ The EAAD model is discussed in more detail in a later section, although Table 1 provides a summary of its underlying assumptions and predicted^30^ kinetic features.

## 4 Results and Discussion

### 4.1 Photocatalyzed Destruction of a Conformal Film of SA Deposited on a Photocatalytic Film: System S1

As noted earlier, system **S1** represents the bulk of the studies of the kinetic studies of reaction 1 carried out to date, in which it is usually found that the kinetics of the removal of a uniform film of SA via reaction 1 is zero order, i.e., they fit eq 12 of the simplified 2D kinetic model.^4,20−26^ Not surprisingly, given its uniform photocatalytic activity, Activ glass is often cited as an example of a photocatalytic film that yields zero-order kinetics for reaction 1.^36^ Since the excellent fit of the simplified 2D kinetic model to the zero-order decay profiles for reaction 1 for real examples of **S1** is well-established,^4,20−26^ it did not merit further repeating in this study.

### 4.2 Photocatalyzed Destruction of a Single Cylindrical SA Island Deposited on Activ: System S2

A single cylindrical SA island, 2 mm in diameter, ca. 160 nm thick, was deposited on a piece of Activ self-cleaning glass and irradiated from above with UVC radiation (254 nm) emitted from two 15 W germicidal lamps (2 mW cm^–2^), during which the sample was removed at regular intervals and photographed under a microscope, and its height, *h*_*t*_, and width, *W*_*t*_, were determined by profilometry.

**Figure 4:**
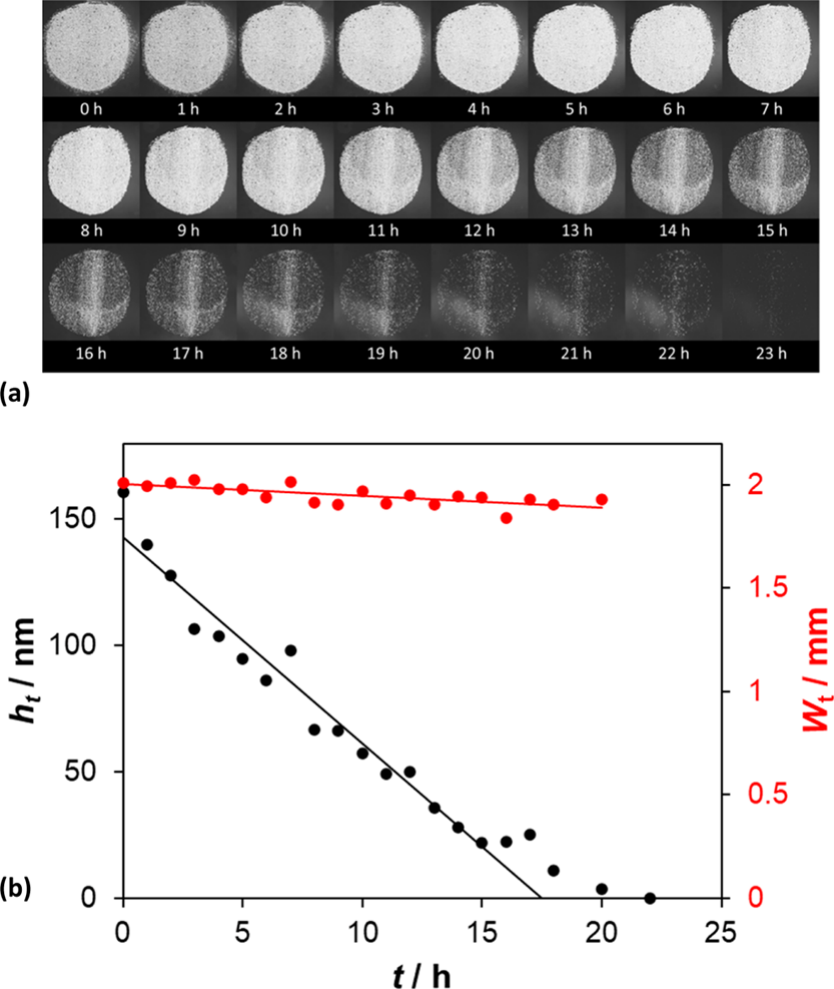
(a) Photographic images recorded using a microscope and (b) thickness, *h*_*t*_, and width, *W*_*t*_, values determined using a profilometer of a single cylindrical SA island on Activ as a function of irradiation time, *t*. The UVC light source used for this work comprised two 15 W germicidal lamps (2 mW cm^–2^).

The optical microscope images recorded of the SA cylindrical SA island as a function *t* are illustrated in Figure 4a and show that with increasing irradiation time, the image of the SA island becomes fainter, presumably due to a decrease in SA film thickness, and that this change was not accompanied by an apparent change in area of the SA island. These features were confirmed by the results of a parallel profilometry study of the same, single SA cylindrical island, illustrated in Figure 4b. The latter results show that −d*h*/d*t* = *c*_*h*_ and −d*W*/d*t* (and so −d*a*/d*t*) ≈ 0, both of which are features predicted by the simple 2D kinetic model, as illustrated by model-predicted *h*_*i*,τ_ vs site, *i*, profiles, for different values of τ, in the insert diagram in Figure 3a and summarized for system **S2** in Table 1.

### 4.3 Photocatalyzed Destruction of an Array of Cylindrical SA Islands Deposited on Activ: System S3

In the previous section, optical microscopy and profilometry were used to validate the simplified 2D kinetic model-predicted features of the kinetics of reaction 1, −d*h*/d*t* = *c*_h_ and −d*a*/d*t* = 0, exhibited by a single cylindrical deposit of SA on a uniform photocatalytic coating. Unfortunately, given the very low FT-IR absorbance of a single cylindrical SA deposit, it was not possible to confirm, for this system, the 2D model-predicted linear decrease in the fractional amount of SA, i.e. *f*_SA,*t*_, as a function of irradiation time, τ (or *t*). However, it was possible to measure both the optical microscope images and the FT-IR absorbance of an array of cylindrical SA islands deposited on Activ as a function of *t*.

**Figure 5:**
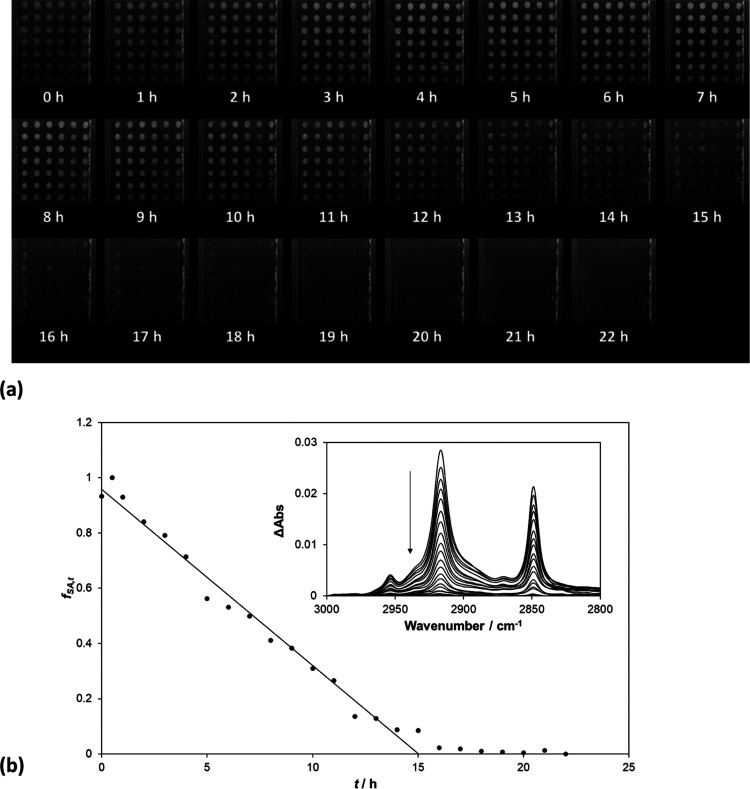
(a) Photographic images recorded as a function of *t* for an array of cylindrical SA islands on Activ and (b) insert plot, FT-IR spectra of the array recorded every 60 min during irradiation, and the main diagram of the fractional integrated absorbance, *f*_SA,*t*_, (=Abs_int_(*t*)/Abs_int_ (*t* = 0) = [SA]_T,*t*_/[SA]_T_,_0_), determined using the spectra in the insert plot, vs *t*. The UVC light source used for this work comprised two 15 W germicidal lamps (2 mW cm^–2^).

Figure 5a illustrates the recorded optical microscope images for an array of cylindrical SA islands on Activ irradiated with UVC as a function of *t*. These images show that, as with a single cylindrical SA island, the appearance of each island gets fainter with increasing *t*, but its area does not change. The insert diagram in Figure 5b illustrates the FT-IR absorbance spectra of the SA cylindrical island array on Activ, recorded at regular intervals during irradiation. At each time, *t*, a value for the integrated absorbance, Abs_int_(*t*), was then calculated from the appropriate absorbance FT-IR spectrum and then used to calculate a value of *f*_SA,*t*_, where *f*_SA,*t*_ = Abs_int_(*t*)/Abs_int_ (*t* = 0), which in turn is equal to the model predicted parameter, ([SA]_T,*t*_/[SA]_T_,_0_). A plot of *f*_SA,*t*_ vs *t* for this system is illustrated in the main diagram of Figure 5b and reveals an excellent fit to zero-order kinetics, in agreement with that predicted by the simplified 2D kinetic model (see Table 1.) The simplified 2D model predicts that the kinetics for the array should be identical to those exhibited by a single cylindrical SA island, and this is confirmed by noting that the *f*_SA,*t*_, vs *t* decay trace for an array illustrated in Figure 5b is nearly identical to that of the *h*_*t*_ vs *t* trace, recorded using a profilometer, for a single cylindrical SA island illustrated in Figure 4b, with half-life, *t*_1/2_, values for these two systems of 7.5 and 8.7 min, respectively.

### 4.4 Photocatalyzed Destruction of a Single Cylindrical SA Island and Array Deposited on a Coating of P25 TiO_2_: Systems S2 and S3

One possible reason for the discrepancy between the kinetics of reaction 1 for a cylindrical SA island on Activ glass, for which, −d*h*/d*t* = *c*_h_ and −d*a*/d*t* = 0 (see Figure 4) and the findings of Ghazzal et al.,^30^ for SA islands on a sol–gel TiO_2_ film, where −d*h*/d*t* = 0 and −d*a*/d*t* = *c*_a_ (see Figure 1), is that in the latter work, the surface activity of the photocatalytic sol–gel film is likely to be much less uniform than that of Activ.

Previous work by this group^28^ has established that while for a uniform film of SA deposited on Activ glass, *n* = 0, when coated on a film of P25 TiO_2_, *n* ≈ 1. The latter effect was due to a significant distribution in surface activity, i.e., a distribution in *k*_*i*_.^10,28^

**Figure 6:**
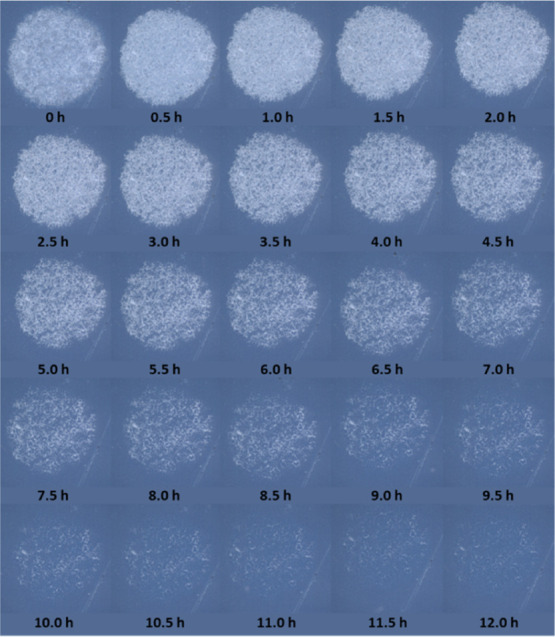
Photographic images recorded using an optical microscope, as a function of irradiation time *t*, of a single cylindrical SA island on a P25 TiO_2_ film. The UVC light source used for this work comprised two 15 W germicidal lamps (2 mW cm^–2^).

To see how reaction site activity distribution affects the kinetics of reaction 1 for an array of cylindrical islands of SA, in this work, a P25 TiO_2_ film was tested under the same conditions as used above for Activ. In the study of system **S2**, the photocatalytic destruction of a single cylindrical SA island, using a P25 film, it was not possible to record the variation in the profile of the SA cylinder because the profiler needle tended to scratch the P25 TiO_2_ film. However, it was possible to monitor the photocatalyzed decay of a SA island on P25 TiO_2_ using optical microscopy, and the results of this work are illustrated in Figure 6. A brief inspection of the images in Figure 6 shows that with increasing *t*, while the SA island largely preserves its circular footprint, −d*a*/d*t* = 0, there is some evidence of photocatalytic activity hot spots on the P25 TiO_2_ film leading to regions of SA within the island perimeter to disappear before the surrounding SA, so that the images of the SA island take on an increasingly pitted, as well as the usual fainter, appearance with increasing *t*.

**Figure 7:**
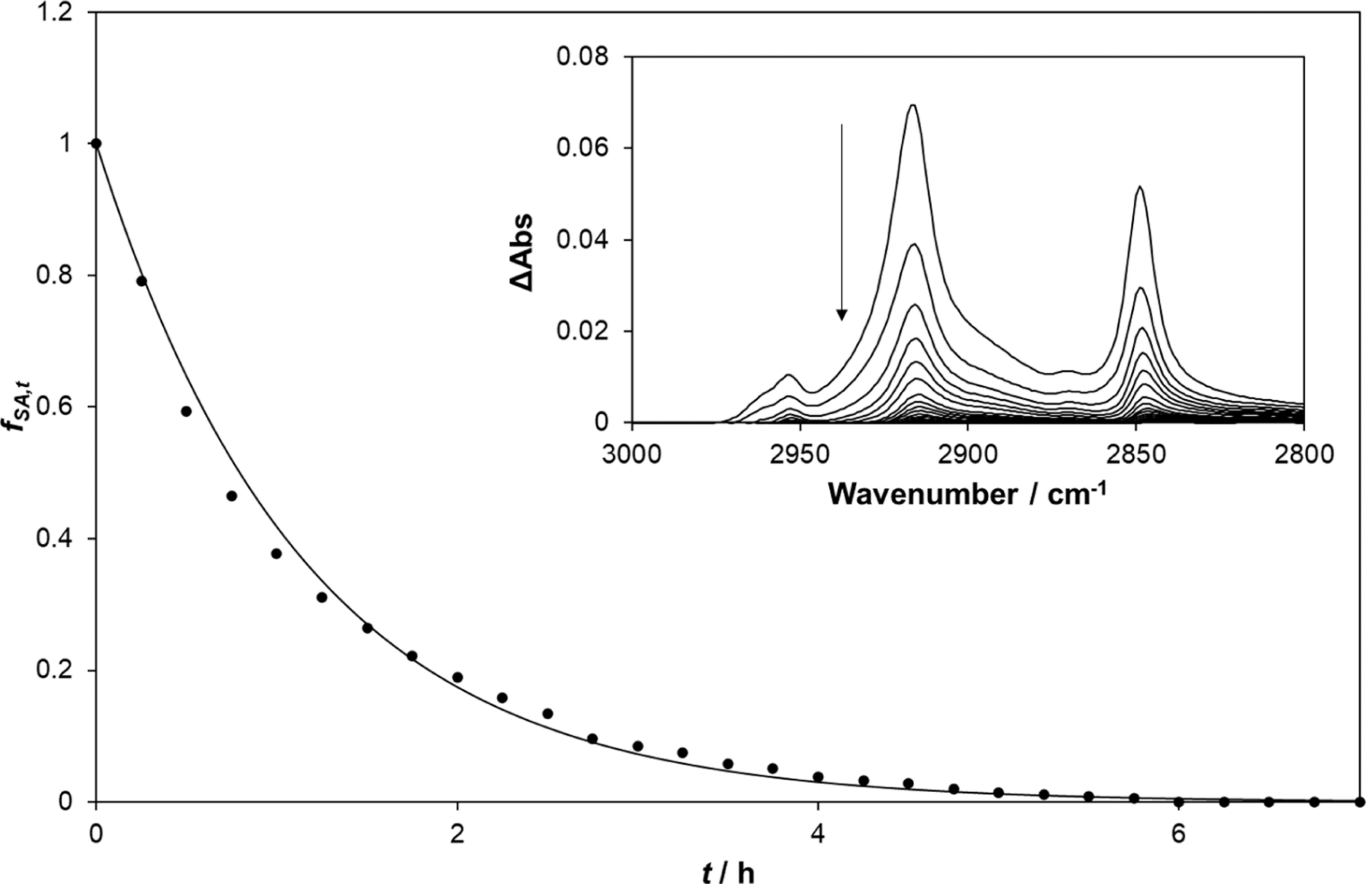
FT-IR spectra of the array recorded every 30 min during irradiation and main diagram of the fractional integrated absorbance, *f*_SA,*t*_, (= Abs_int_(*t*)/Abs_int_ (*t* = 0) = [SA]_T,*t*_/[SA]_T_,_0_), determined using the spectra in the insert plot, vs *t*. The UVC light source used for this work comprised two 15 W germicidal lamps (2 mW cm^–2^).

In a subsequent study, the FT-IR spectrum of an array of cylindrical SA islands, i.e., system **S3**, on a P25 TiO_2_ film was recorded as a function of *t*, and the results of this work are illustrated in the insert plot in Figure 7. The data in this plot were used to calculate the variation in *f*_SA,*t*_, (= Abs_int_(*t*)/Abs_int_ (*t* = 0) = [SA]_T,*t*_/[SA]_T_,_0_), as a function of *t*, the results from which are illustrated in the main diagram in Figure 7. The latter plot shows that the kinetics of reaction 1 for an array of cylindrical SA islands on a film of P25 TiO_2_ are of a much higher order, i.e., *n* ≈ 1, than those observed when using Activ glass (see Figure 5), where *n* ≈ 0.

As noted earlier, in a previous paper,^28^ such a deviation from zero-order kinetics for a film of SA on a P25 TiO_2_ photocatalytic coating was ascribed to a significant dispersion in surface activity, the presence of which is supported by the reaction hot spots in the optical micrographs illustrated in Figure 6. However, the optical micrograph images illustrated in Figure 6 also show that despite clear evidence of a dispersion of photocatalytic activity across the surface of the P25, the area of the cylindrical islands did not change with increasing *t*, i.e., −d*a*/d*t* = 0. These observations are consistent with the 2D general kinetic model and not the EAAD model and the observation of Ghazzal et al.^30^ that −d*a*/d*t* = *c*_a_.

### 4.5 Photocatalyzed Destruction of a Single SA Volcano: System S4

In the above work, strenuous efforts were made in the production of the SA islands to ensure that they were of uniform thickness, so that not only the average initial thickness of each SA island was the same, *h*_0_, but also, within each island, there was no variation in SA film thickness, *h*_*i*_ = *h*_0_ at all *i*. Thus, in this work, each SA island produced has a cylindrical shape, as illustrated in Figure S1 in the Supporting Information. In contrast, Ghazzal et al.^30^ produced their SA islands by dip-coating the sol–gel TiO_2_ film in a methanolic solution of SA, thereby creating an array of different sized (i.e., different *a*_0_ and *h*_0_) and shaped islands, as illustrated by the optical micrograph outlines in Figure 1. In the latter work, the height of each island was assessed by the gray scale of a photograph of the micrograph, which, for reasons to be discussed later, appears at best a very crude scale.^30^ If the method of assessing height was suspect, what if the SA islands produced by Ghazzal et al. were in fact volcano like in height profile and not table-topped?^30^ Could such a situation, which is by no means unlikely, explain the observation that −d*a*/d*t* = *c*_*a*_, and the possibly doubtful claim that −d*h*/d*t* = 0, the combination of which is the basis of the EAAD kinetic model (see Table 1)?

**Figure 8:**
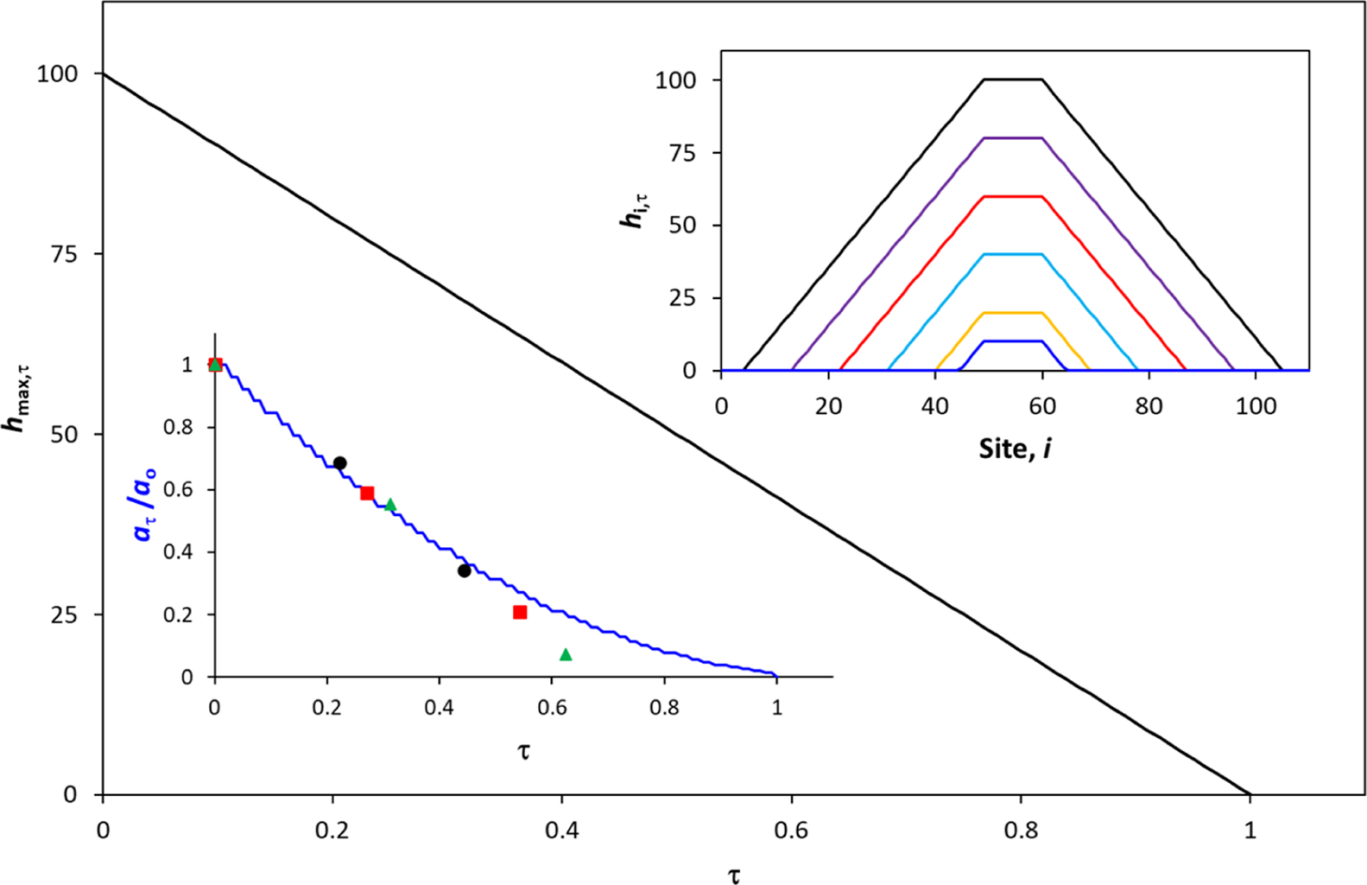
2D kinetic model-predicted decay curves for the photocatalyzed destruction of a “volcano” shaped island of SA on a photocatalytic film of uniform activity. The insert plot on the top right-hand side illustrates the predicted variation in *h*_*i*,τ_ vs *i* profiles at irradiation times, τ, of (from top to bottom) 0, 0.2, 0.4, 0.6, 0.8, and 0.9, respectively. The main diagram and bottom left-hand side insert plots are the predicted variations in *h*_max,τ_ and normalized area, *a*_τ_/*a*_0_, vs τ, respectively. The data points were derived for the large (●), medium (■), and small (▲) SA islands, using the three data sets in Figure 1.

The kinetics of the photocatalytic destruction of a “volcano” shaped island of SA, on a TiO_2_ coating of uniform activity, are readily predicted using the general 2D kinetic model, eq 11, with the usual assumption that all the photocatalytic sites are equally active, β_*i*_ = 1, but with a varying value of α_*i*_, so as to create a volcano-shaped 2D profile. The results of this work are illustrated in Figure 8, with the top right-hand side insert plot showing the predicted variation in the *h*_*i*,τ_ vs site number, *i*, profile for an initially volcano-shaped profile, with increasing irradiation times, τ, from which it is clear that both the maximum thickness of the SA island, *h*_max,τ_ and the width/diameter, *W*_τ_, decrease linearly with increasing τ, i.e., −d*h*/d*t* = *c*_*h*_ and −d*W*/d*t* = constant. However, as the SA “volcano” will be circular in its 3D form, its area, *a*, is related to *W* via the expression, *a* = π(*W*/2)^2^, so that that −d*a*/d*t* will be >0 but not constant, as it will decrease with increasing *t*. The general 2D kinetic model-predicted non-linear decay in the normalized island area (*a*_τ_/*a*_0_) as a function of irradiation time τ is illustrated in the bottom left-hand side insert plot in Figure 8.

Thus, according to the general 2D kinetic model, although a volcano-shaped island of SA would appear to shrink with increasing *t*, as observed by Ghazzal et al.,^30^ it would not shrink at a constant rate, and would simultaneously appear to fade, as −d*h*/d*t* = *c*_h_. These predictions do not appear to help explain the kinetic features reported by Ghazzal et al.^30^ for their SA islands, namely −d*a*/d*t* = constant, *c*_*a*_, and −d*h*/d*t* = 0, which form the basis of the EAAD model.

In order to test the 2D kinetic model predictions for a volcano-like SA island, such an island was prepared on a sol–gel TiO_2_ coating. The latter photocatalytic coating was used because it was sufficiently active that it could remove the SA islands within a 30 h irradiation period using 365 nm radiation, which Activ was unable to do because of its very thin TiO_2_ coating, ca. 15 nm. In addition, unlike the P25 TiO_2_ coating, the sol–gel TiO_2_ coating was sufficiently robust that it was not damaged by the needle of the profilometer. The experiment was also restricted to using 365 nm radiation, and not 254 nm, by the transmission characteristics of the fiber optic cable that needed to be used, in order to allow in situ irradiation of the SA volcano on the sol–gel TiO_2_ coating in the profilometer.

**Figure 9:**
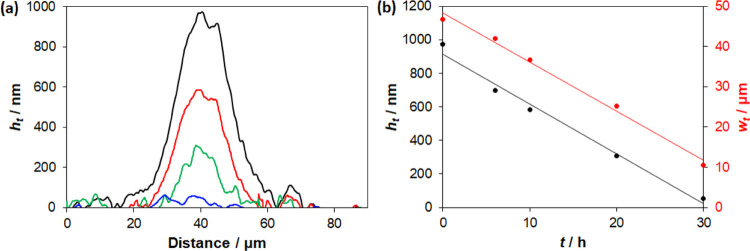
(a) Plot of the profile of a SA volcano-shaped island as a function of irradiation time, *t*, where *t* was (from top to bottom) 0, 10, 20, and 30 h, respectively; (b) based on the data in (a), subsequent plot *h*_max_ and *W* as a function of *t*. The irradiation was performed in situ in the profilometer using a fibre optic deliverying 365 nm radiation, with an irradiance of 80 mW cm^–2^.

The volcano-shaped island of SA on a sol–gel TiO_2_ coating was then monitored by profilometry as the TiO_2_ coating was irradiated with 365 nm radiation delivered via a fiber optic cable. The variation in the profile of the SA island as a function of irradiation time is illustrated in Figure 9a and appears to confirm the 2D general model predictions that both the area and the maximum thickness will decrease with increasing *t*. The profiles illustrated in Figure 9a were used to construct plots of maximum SA film thickness, *h*_max_, and SA island width, *W*, as a function of *t*, the results of which are illustrated in Figure 9b. These results show that, as predicted by the general 2D kinetic model, both the maximum thickness of the SA island, *h*_max,*t*_ and the width/diameter, *W*_*t*_, decrease linearly with increasing *t*, i.e., −d*h*/d*t* = *c*_h_ and −d*W*/d*t* = constant.

## 5 The EAAD Model

As noted earlier, in their study of reaction 1, using islands of SA, Ghazzal et al.^30^ reported −d*a*/d*t* = constant, *c*_a_, and −d*h*/d*t* = 0 and rationalized these findings using an EAAD kinetic model, of which the underlying assumptions and predicted kinetic features are given in Table 1. It should be noted however, that in this model it is not at all obvious why the rate of reaction depends upon the area of the island, rather than its circumference, given the proposed mechanism relies on edge attack from “radical species formed throughout the uncovered surface and diffusing toward the islands edges”.^30^

Since the model assumes that the rate depends on the presence of an uncovered part of the photocatalytic surface, it follows that it will be zero when the photocatalytic film has a conformal coating of SA, i.e., system **S1**. This prediction of the EAAD model of no reaction, when the photocatalytic film is completely covered with SA, is clearly at odds with the fact that it has never been observed, and instead that for all examples of **S1**, the rate is >0.^4,20−26^ Support for the EAAD model is further weakened by the fact that none of the results reported here support the predictions of the EAAD model; rather, all the data fit the predictions made by the 2D kinetic model in which only occupied sites are active and no preferential edge attack occurs.

If the EAAD kinetic model is not valid and the kinetics of SA islands are described only by the 2D kinetic model, what then could explain the observations that Ghazzal et al.^30^ reported, −d*a*/d*t* = constant, *c*_a_, and −d*h*/d*t* = 0? One possibility is that in the latter work, the islands of SA were volcano-shaped and not table-topped. We have seen that the 2D kinetic model predicts that such islands will shrink (and fade) with increasing *t*, and these predictions were confirmed in our profilometer study of such an island (see Figure 9). Support for the proposal that the SA islands studied by Ghazzal et al.^30^ are volcano-shaped comes from the observation that the 2D general kinetic model-predicted decay in *a*_τ_/*a*_0_, illustrated in Figure 8 for a volcano-shaped island, provides a good fit to most of the *a*_*t*_ vs *t* data reported by Ghazzal et al.^30^ for the three SA islands illustrated in Figure 1, as shown by the data points in the plot in Figure 8.^30^ This fit is impressive, given that some deviation from the model would be expected since the model-predicted *a*_τ_/*a*_0_ vs τ plot is for a symmetrical volcano-shaped SA island on a photocatalytic film of uniform activity, whereas the data points derived from Figure 1, are for three, decidedly asymmetrically shaped, SA islands.

It would appear, therefore, that the major sticking point in reconciling the work of Ghazzal et al.^30^ and the 2D kinetic model and all the results reported here is their observation that −d*h*/d*t* = 0, i.e., their claim that the islands shrink but apparently do not fade with increasing *t*. The latter observation lies counter to what we observed for volcano-shaped SA islands, as illustrated by the results in Figure 9, where −d*h*/d*t* = *c*_h_, as predicted by the general 2D kinetic model. Could it be that the gray-scale optical microscopy method that Ghazzal et al.^30^ used to measure SA island height was not up to the task and that in fact −d*h*/d*t* was ≥0? Certainly, Ghazzal et al.^30^ note that the dark and light gray levels of their optical micrographs lie in the very broad-thickness regions of 120–80 nm and <80, respectively. It follows that the error associated with the gray scale measurement of SA island height will be large. More striking evidence that optical microscopy is poorly suited for measuring *h* is obtained by comparing real height, as measured by AFM, and inverted gray scale “height” using data reported by Ghazzal et al.^30^ for a line of SA, as illustrated by the results of such a comparison described in S2 in the Supporting Information. Taken together, the results cast doubt on the veracity of the original claim^30^ that −d*h*/d*t* = 0. Thus, in the work of Ghazzal et al.,^30^ if −d*h*/d*t* was in fact >0, then this, coupled to the remaining observation that −d*a*/d*t* = *c*_a_ would be entirely consistent with the 2D kinetic model for volcano-shaped SA islands, which will shrink and fade with irradiation.

## 6 Conclusions

The removal of a cylindrical SA island, or an array of islands, via reaction 1, mediated by a photocatalytic film, such as Activ self-cleaning glass, or a P25 TiO_2_ coating on glass, results in a decrease in their thickness, *h*, with *t*, but with d*a*/d*t* = 0, i.e., the islands fade but do not shrink. This is opposite to what Ghazzal et al.^30^ reported for SA islands on a sol–gel TiO_2_ film, namely, they shrink, rather than fade, i.e., d*h*/d*t* = 0 and −d*a*/d*t* = *c*_a_. However, the latter observation of shrinking SA islands is observed when the SA islands are more volcano-like in profile, rather than cylindrically-shaped; however, these volcano-like SA islands also fade, i.e., −d*h*/d*t = c_h_*. A simple 2D kinetic model can be used to rationalize the results of this work, and to fit those from a previous study on the removal of SA islands using a sol–gel TiO_2_ film,^30^ in which −d*a*/d*t* = *c*_a_, but not to the additional claim that −d*h*/d*t* = 0, although the validity of the latter is questionable, given the demonstrably poor ability of the optical gray scale used to assess *h*.

The above findings suggest that it is likely that all the active photocatalytic sites on a self-cleaning film, covered with a pollutant such as SA, will be effective in destroying it, so that with islands of pollutants, its destruction at the edges will be no faster than in the bulk. It is suggested here that any apparent shrinking of a pollutant island with *t* is due to its thickness profile being more like a volcano rather than a cylinder.

## Supporting Information Available

Schematic images of a cylindrical island and array of such islands based on the 2D kinetic model, and comparison of AFM and optical microscope height measurements (PDF)

## Notes

All data are provided in full in the Results section of this paper and the Supporting Information accompanying this paper.
