# Supplementary material for: Study and Modeling of the Kinetics of the Photocatalytic Destruction of Stearic Acid Islands on TiO2 Films
Source: J Phys Chem C Nanomater Interfaces. 2023 Jun 20;127(25):12194–205. doi: 10.1021/acs.jpcc.3c00952 (PMC10316406; doi:10.1021/acs.jpcc.3c00952)
Supplement: Supplementary file 1 — jp3c00952_si_001.pdf [file jp3c00952_si_001.pdf]

*Electronic Supplementary Information (ESI)

Study and modeling of the kinetics of the photocatalytic destruction of Stearic Acid (SA) islands on TiO_2 films

*Saleh Alofi, Christopher O'Rourke and Andrew Mills**

School of Chemistry and Chemical Engineering, Queens University Belfast, Stranmillis Road, Belfast, BT9 5AG, UK

e-mail: andrew.mills@qub.ac.uk

S1. Predicted images of a cylindrical island and array of such islands based on the 2D kinetic model

The 2D kinetic model is based on a line of 100 photocatalytic sites, *i,* each with the same zero-order rate constant, k_i, with an initial covering of SA of thickness, $h_{i,o}$, which is assumed to be 100 units (arbitrary units) high/thick. If this 2D sheet of SA is rotated about its central axis, then a cylindrical island of SA is generated, as illustrated in Figure s1. Since the photocatalytic film has a uniform activity, it follows that the kinetic features predicted by the 2D model for a SA sheet, will also be those for the 3D cylindrical island illustrated in Figure S1 and a 3D array of such (identical) islands, as illustrated in Figure S2. The scales used in Figure S2, have been chosen so that they more represent the actually array of SA cylinders prepared in this work, i.e. are ca. 150 nm thick and 2 mm diameter.

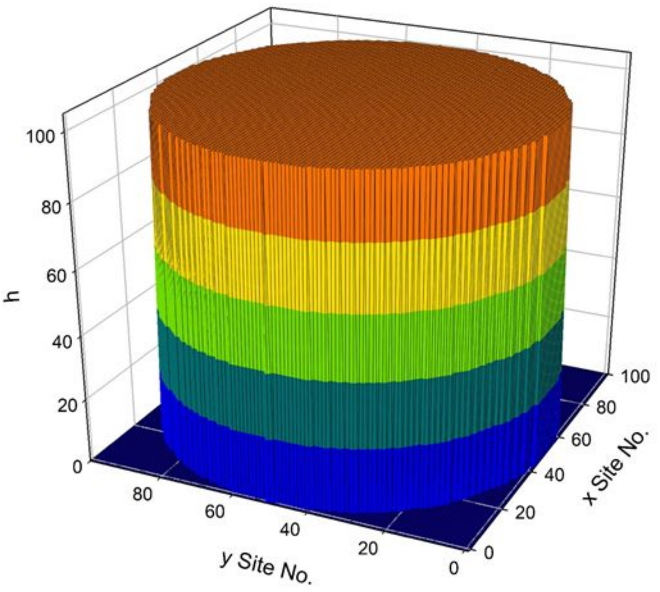

Figure S1. Plot a cylindrical island of SA as a function of reaction site number in the *x* and *y* planes, with each site occupied by a SA film with an initial thickness value, $h_{i,o}$, of 100 (arbitrary units);

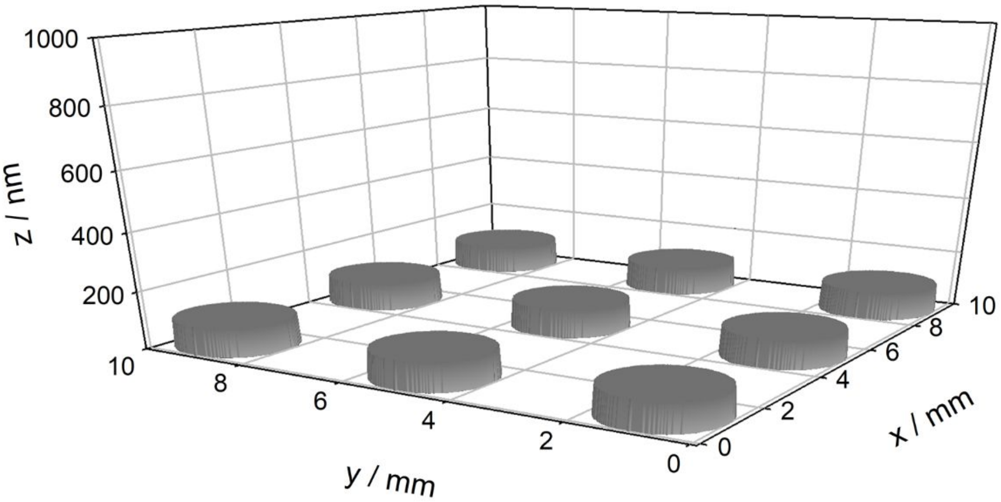

Figure S2. Plot an initial array of identical cylindrical islands (typically 150 nm thick and 2 mm diameter) of SA of the sort illustrated in Figure 1, coating a photocatalytic film of uniform activity.

S2. Comparison of AFM and optical microscopy images and heights of SA deposits

Ghazzal et al.[1] reported an AFM and optical microscopy image of the same SA deposits, including a plot of the variation of *h* (as measured by AFM) and inverted gray scale value along the same horizonal (*x*- scale) line. The results of this work are illustrated in Figure S3.

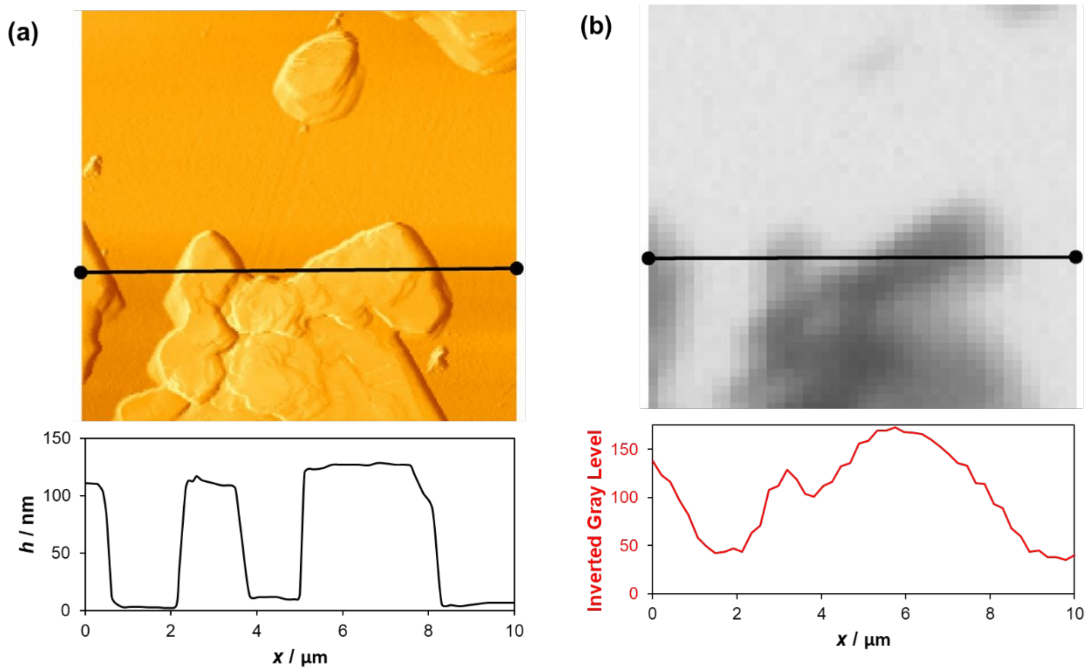

Figure S3. (a) Representative 10 µm x 10 µm AFM image of the SA-coated titania film the height profile below corresponding to the solid horizontal line on the image. **(b)** Optical microscopy image of the same region of the SA-coated titania film with the inverted gray level profile corresponding to the solid horizontal line at exactly the same location as the line in (a). Adapted with permission from ref [1]. Copyright 2011 Elsevier.

The AFM (real) height profile in Figure S3(a) was superimposed on the inverted gray scale profile of the same line of SA and the results are illustrated in Figure S4(a). Figure S4(a) reveals that the AFM height profile of the SA deposit is very different to that indicated by the inverted grey scale of the optical microscopy. Indeed, Figure 4(a) shows that there are clear areas where the SA doesn't exist and yet the gray scale value suggest it does and that its height is significant.

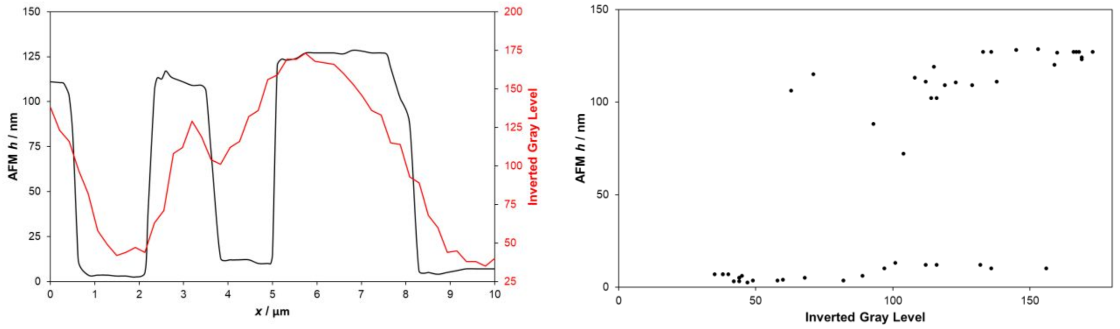

(a) **(b)**

Figure S4. (a) Reported AFM (black) and inverted gray scale (red) profiles for the same horizonal line (*x* scale) through a series of SA deposits and (b) a plot of the data in (a) in the form of *h*, as measured by AFM vs inverted gray scale value at the same vale of *x*, from 0 to 10 μm.

Figure S4(b), a plot of AFM *h* value vs inverted gray scale value for all values of *x* in Figure 10(a), underlines the substantial lack of correlation between the actual height, as measured by AFM, and the height suggested by the inverted gray scale value. The poor ability of optical microscopy to measure *h*, undermines the claim that -d*h*/d*t* = 0, and suggests that the SA islands studied were more likely volcano-, rather than table-topped mountain shaped.

References

(1) Ghazzal, M. N.; Barthen, N.; Chaoui, N. Photodegradation Kinetics of Stearic Acid on UV-irradiated Titania Thin Film Separately Followed by Optical Microscopy and Fourier Transform Infrared Spectroscopy. *Appl. Catal. B* **2011**, 103, 85–90.
